# Supplementary material for: FBXO22 degrades nuclear PTEN to promote tumorigenesis
Source: Nat Commun. 2020 Apr 6;11:1720. doi: 10.1038/s41467-020-15578-1 (PMC7136256; doi:10.1038/s41467-020-15578-1)
Supplement: Supplementary file 2 — Supplementary Information [file 41467_2020_15578_MOESM2_ESM.pdf]

Supplementary Information

FBXO22 Degrades Nuclear PTEN to Promote Tumorigenesis

Ge *et al.* 2020

Supplementary Figures

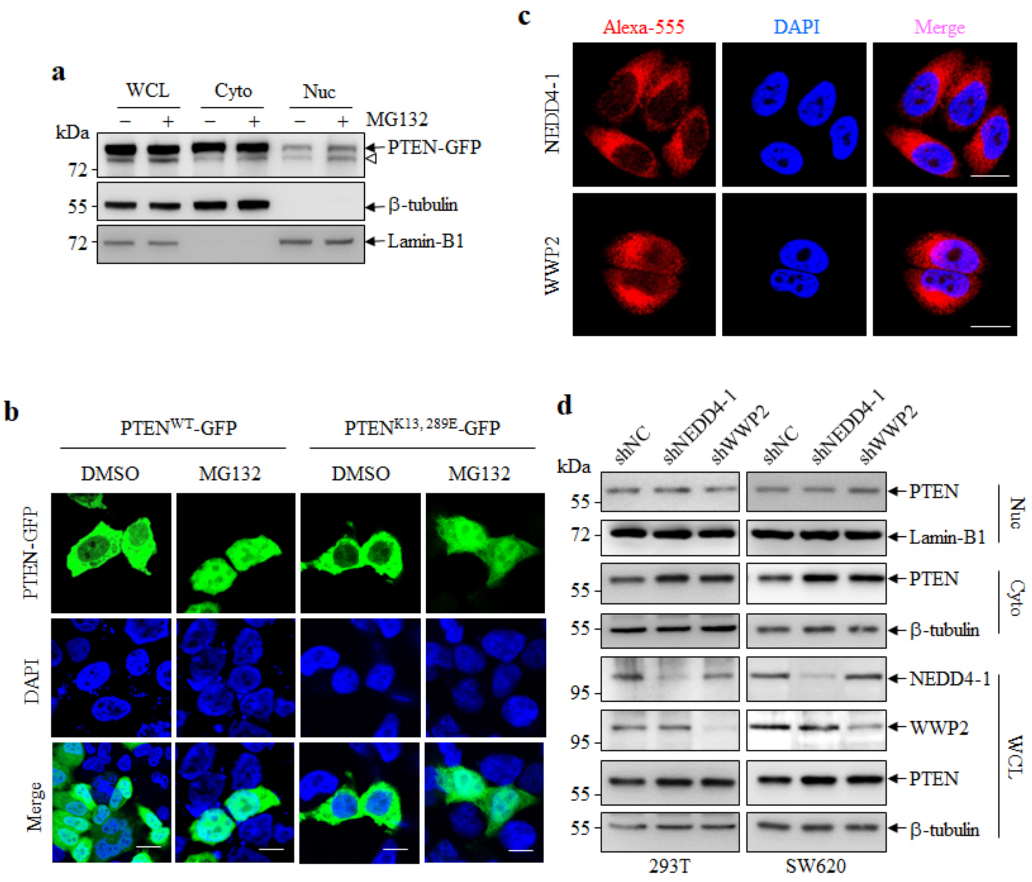

Supplementary Figure 1. NEDD4-1 and WWP2 do not regulate the abundance of nuclear PTEN.

(**a**) Western blots of PTEN-GFP in the cytoplasmic (Cyto) and nuclear (Nuc) fractions as well as whole cell lysates (WCL) of 293T-PTEN^{KO} cells treated as in Fig. 1**a**. The empty triangle points to an unknown band.

(**b**) 293T-PTEN^{KO} cells were transfected with PTEN^{WT}-GFP or PTEN$^{K13, 289E}$-GFP for 8 hours, followed by treatment with 10 μM MG132 or DMSO for 6 hours, stained with DAPI, and imaged with confocal microscopy. Scale bar represents 10 μm.

(**c**) Representative images of immunofluorescent staining of NEDD4-1 and WWP2 respectively with anti-NEDD4-1 and WWP2 antibodies together with re-staining of DAPI in Hela cells. Scale bar represents 20 μm.

(**d**) Western blots of indicated proteins in the cytoplasmic (Cyto) and nuclear (Nuc) fractions as well as whole cell lysates (WCL) of 293T and SW620 cells transduced by retroviruses encoding shRNAs targeting NEDD4-1 (shNEDD4-1) and WWP2 (shWWP2), along with shNC used as a negative control.

The experiments shown in **a-d** were repeated three times with similar results, and the results of one representative experiment are shown. Source data are provided as a Source Data file.

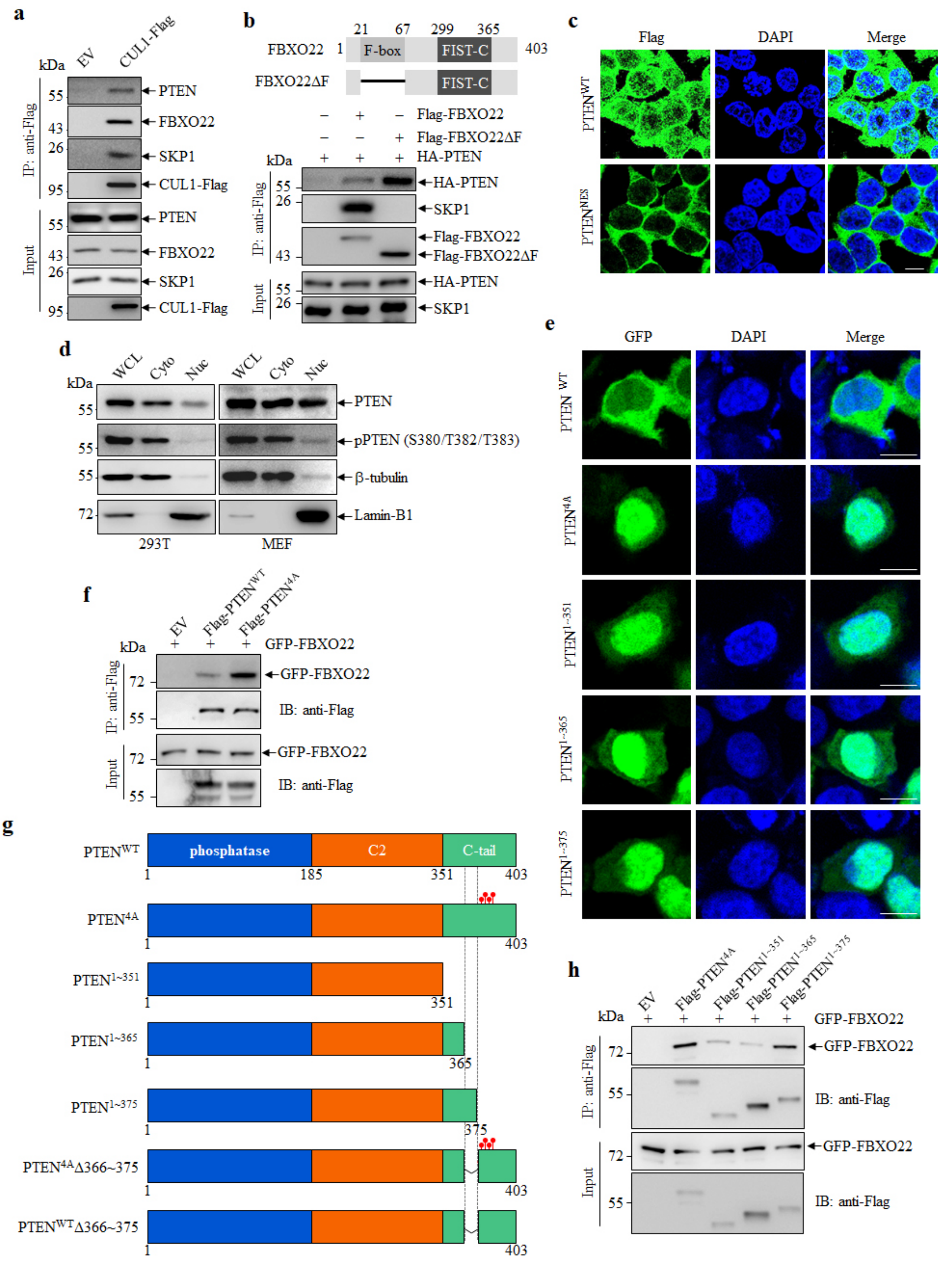

Supplementary Figure 2. FBXO22 and PTEN derivatives.

(**a**) 293T cells transfected with Flag-tagged CUL1 were subjected to immunoprecipitation with an anti-Flag antibody. Western blots of indicated proteins in the input and immunoprecipitates are shown.

(**b**) Top, schematics of FBXO22 and its F-box-deleted mutant FBXO22ΔF. Bottom,

Flag-tagged FBXO22 or FBXO22ΔF were co-transfected with HA-tagged PTEN into 293T cells, followed by immunoprecipitation with anti-Flag antibody and Western blot for indicated proteins.

(**c**) Representative images of immunofluorescent staining of Flag-tagged proteins with an anti-Flag antibody together with re-staining of DAPI in 293T cells transfected with Flag-tagged $PTEN^{WT}$ or $PTEN^{NES}$. Scale bar represents 10 μm.

(**d**) Western blots of indicated proteins in cytoplasmic (Cyto) and nuclear (Nuc) fractions as well as whole cell lysates (WCL) of 293T cells and MEF.

(**e**) Representative images of GFP-tagged PTEN derivatives transfected in 293T cells with re-staining of DAPI. Scale bar represents 10 μm.

(**f**) 293T cells co-transfected with GFP-FBXO22 and Flag-tagged $PTEN^{WT}$ or $PTEN^{4A}$ were subjected to immunoprecipitation with an anti-Flag antibody. Western blots of Flag- and GFP-tagged proteins in the input and immunoprecipitates are shown.

(**g**) Schematics of PTEN derivatives.

(**h**) 293T cells co-transfected with GFP-FBXO22 and Flag-tagged PTEN derivatives were subjected to immunoprecipitation with an anti-Flag antibody. Western blots of Flag- and GFP-tagged proteins in the input and immunoprecipitates are shown.

All experiments except **g** were repeated three times with similar results, and the results of one representative experiment are shown. Source data are provided as a Source Data file.

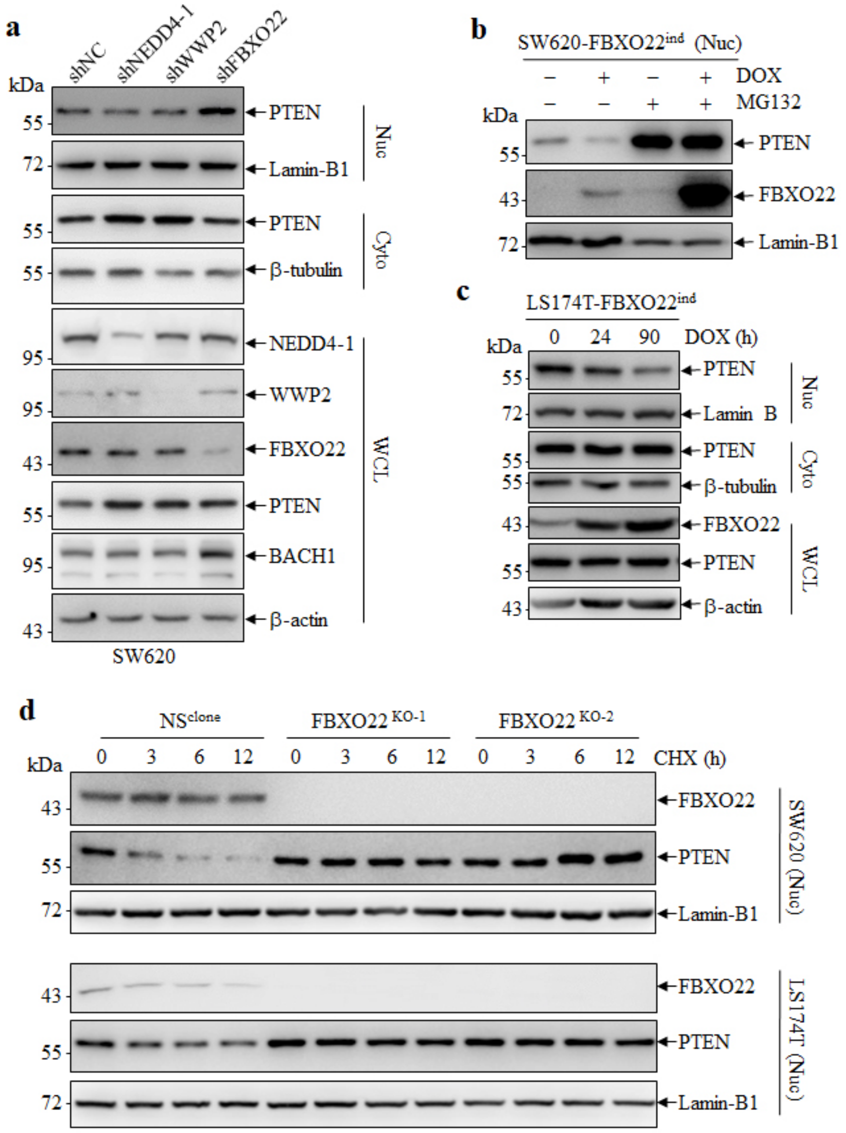

Supplementary Figure 3. FBXO22 downregulates nuclear but not cytoplasmic PTEN.

(**a**) Western blots of indicated proteins in the cytoplasmic and nuclear fractions as well as whole cell lysates of 293T cells transduced by retroviruses encoding shRNAs targeting FBXO22, NEDD4-1 and WWP2, along with shNC used as a negative control.

(**b**) SW620-FBXO22^{ind} cells with or without 600 ng/ml DOX administration for 60 hours were treated with 10 μM MG132 for 6 hours, followed by Western blot analysis of indicated proteins in the nuclear fractions.

(**c**) A DOX-inducible expression system encoding FBXO22 was introduced by lentiviruses into LS174T cells (LS174T-FBXO22^{ind}). Western blots of indicated

proteins in the nuclear and cytoplasmic fractions as well as whole cell lysates of LS174T-FBXO22^{ind} cells by 600 ng/ml DOX administration for indicated times.

(**d**) Western blots of indicated proteins in the nuclear fractions of two clonally derived SW620 (top) and LS174T (bottom) cell lines depleted of FBXO22 (FBXO22^{KO-1} and FBXO22^{KO-2}) and a negative control (NS^{clone}) respectively treated with 50 μg/ml CHX for hours as indicated.

All experiments were repeated three times with similar results, and the results of one representative experiment are shown. Source data are provided as a Source Data file.

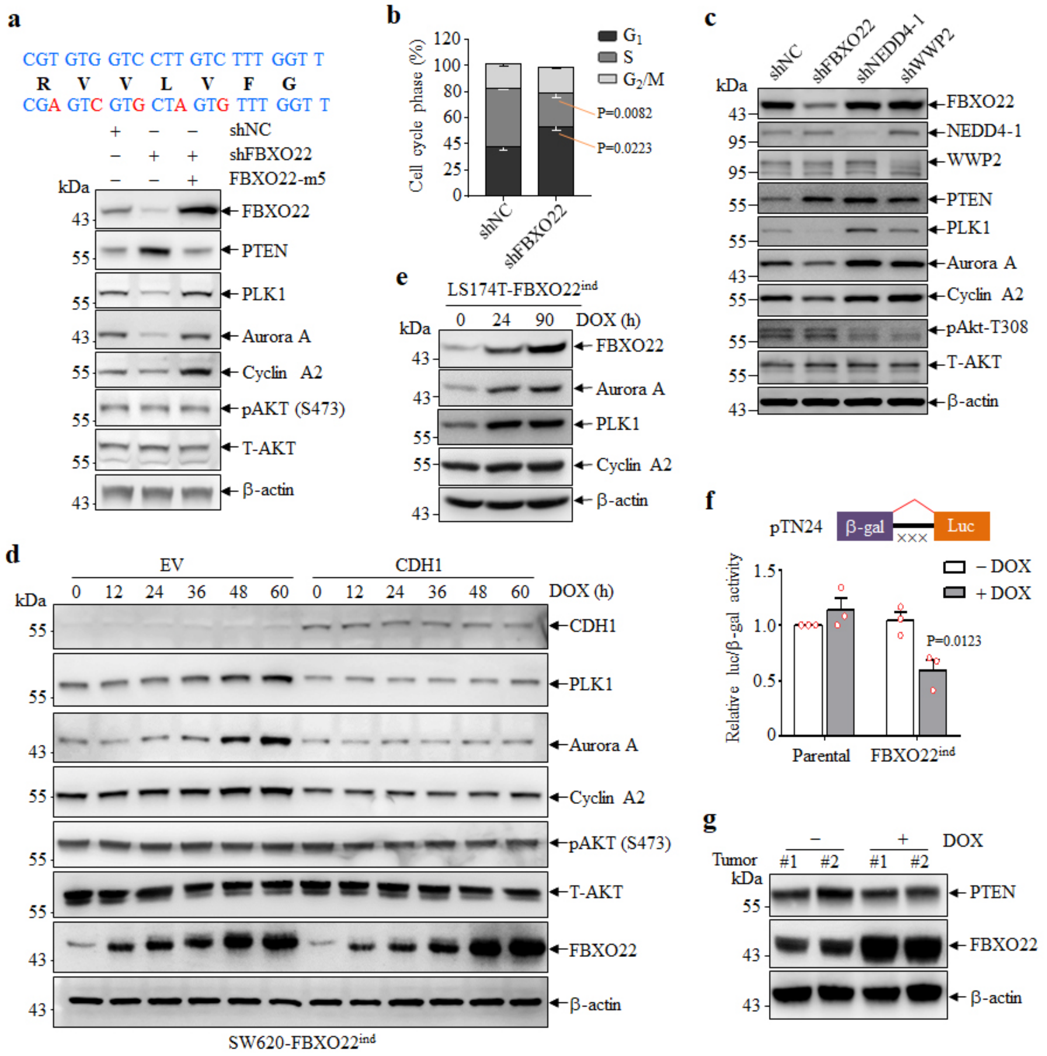

Supplementary Figure 4. FBXO22 regulates APC/C–CDH1 substrates.

(**a**) A shRNA-resistant version of FBXO22 with the shRNA target sequence mutated as illustrated was re-expressed in FBXO22-knocked down SW620 cells by lentiviruses (top), and Western blots of indicated proteins were shown (bottom).

(**b**) SW620 cells transduced by retroviruses encoding shFBXO22 or shNC were stained with PI to analyze the cell cycle distribution of each cell type by flow cytometry, and cell number % of each cell cycle phase relative to total phases was shown.

(**c**) Western blots of indicated proteins in SW620 cells transduced by retroviruses encoding shRNAs targeting FBXO22 (shFBXO22), NEDD4-1 (shNEDD4-1) and WWP2 (shWWP2) along with shNC as negative control.

(**d**) SW620-FBXO22^{ind} cells were transduced by lentiviruses encoding CDH1 or EV, treated with DOX for indicated times, and subjected to Western blots for indicated

proteins.

(**e**) Western blots of indicated proteins in LS174T-FBXO22^{ind} cells by 600 ng/ml DOX administration for indicated times.

(**f**) The pTN24 minigene construct (top) consisting of β-gal, an upstream intron that contains three translational stop codons (represented as '×××') and luciferase (Luc), was transfected into SW620-FBXO22^{ind} cells with or without FBXO22 induction by DOX administration, and the ratios of luciferase expression relative to β-gal expression are shown (bottom).

(**g**) Western blots of indicated proteins in 2 of the tumors randomly selected from each group in Figure 4j.

The experiments shown in **a**, **c**-**e** and **g** were repeated three times with similar results, and the results of one representative experiment are shown. Each data point in panel **b** and the data in panel **f** are presented as the mean ± SEM, n = 3 independent experiments; two-tailed unpaired *t*-test for panel **b** and **f**. Source data are provided as a Source Data file.

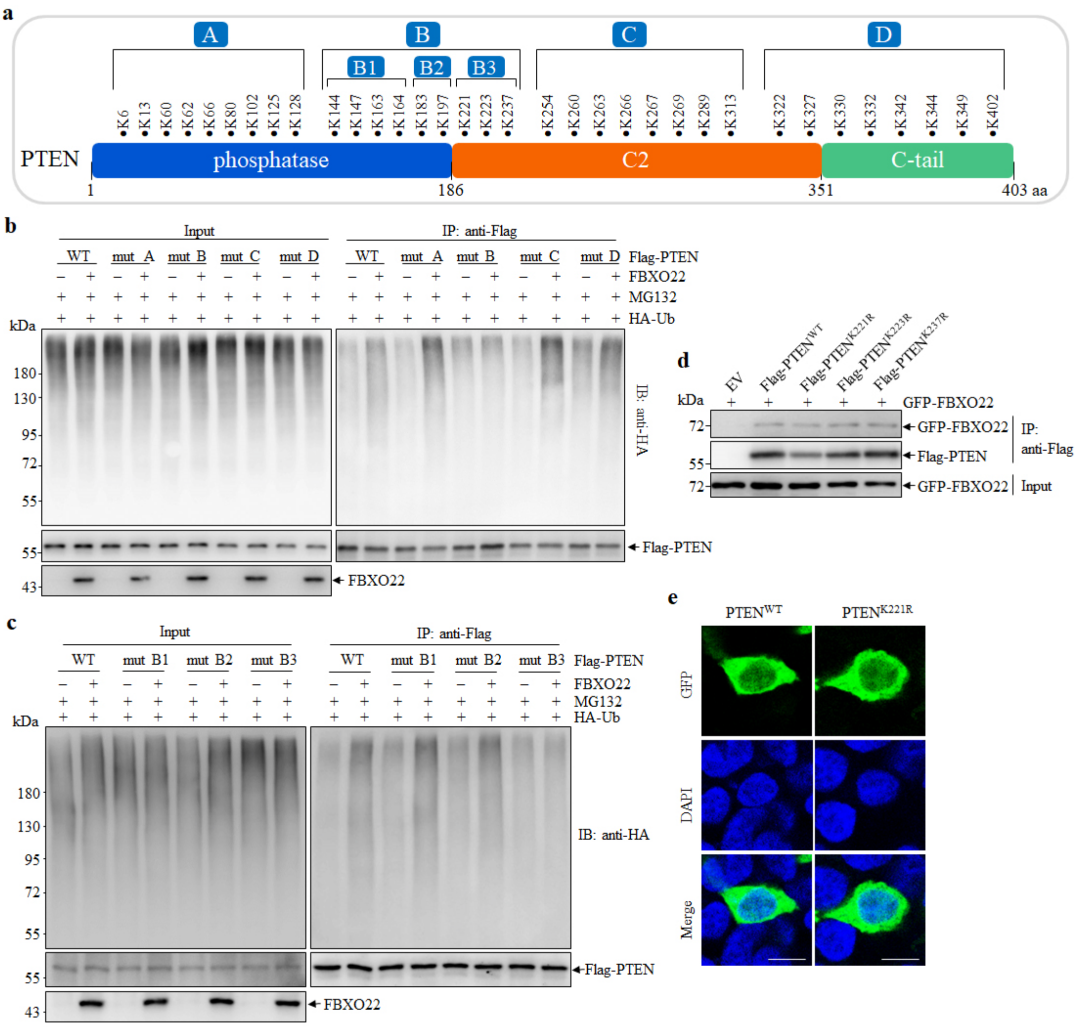

Supplementary Figure 5. Identification of ubiquitylated site(s) of PTEN by FBXO22.

(**a**) Schematic representation of PTEN protein domains and grouping strategy of all the lysine residues in PTEN protein sequence.

(**b**, **c**) 293T cells transfected with the indicated plasmids in the first step (**b**) and second step (**c**) of stepwise mutation were treated with 10 μM MG132 for 6 hours, harvested, and submitted to in vivo ubiquitination assay, followed by Western blot analysis.

(**d**) 293T cells co-transfected with GFP-FBXO22 and Flag-tagged PTEN derivates were subjected to immunoprecipitation with an anti-Flag antibody. Western blots of Flag- and GFP-tagged proteins in the immunoprecipitates are shown.

(**e**) Representative images of GFP-tagged PTEN^{WT} or PTEN^{K221R} transfected in 293T cells with re-staining of DAPI. Scale bar represents 10 μm.

All experiments except **a** were repeated three times with similar results, and the

results of one representative experiment are shown. Source data are provided as a Source Data file.

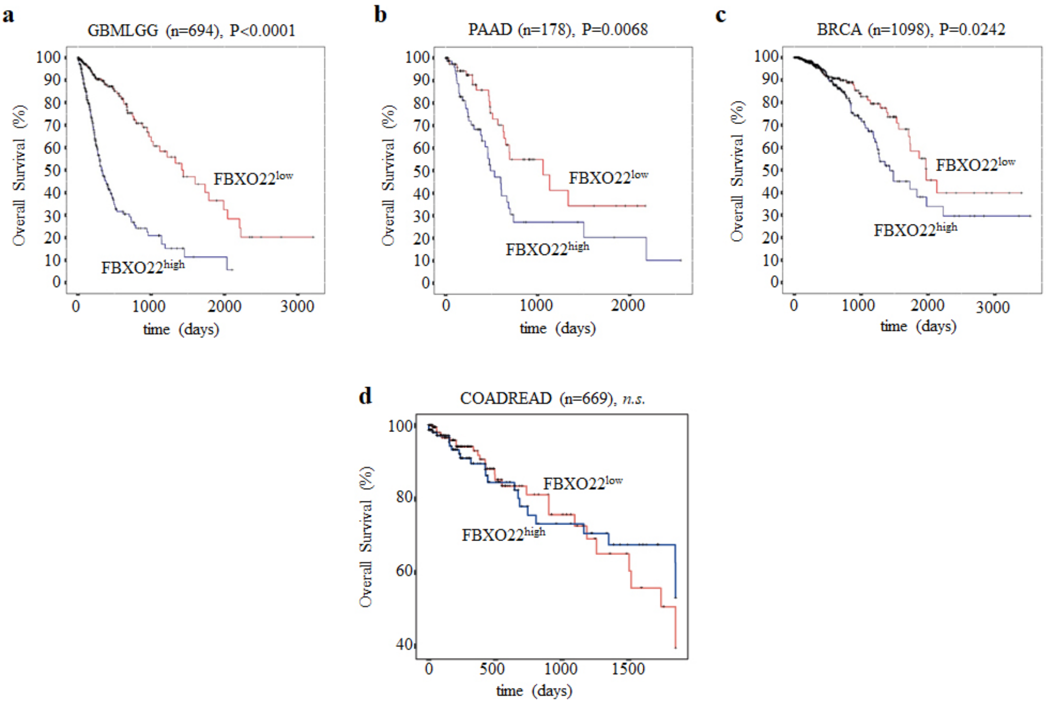

Supplementary Figure 6. FBXO22 predicts poor prognosis in TCGA datasets of some cancer types.

(**a**-**d**) Kaplan–Meier estimates of overall survival of patients respectively with 35% highest and 35% lowest FBXO22 expression in TCGA glioma (GBMLGG, **a**), pancreatic adenocarcinoma (PAAD, **b**), invasive breast cancer (BRCA, **c**), and colorectal cancer (COADREAD, **d**) datasets. Statistical significance was determined by two-sided Mann–Whitney *U* test and P value is shown. *ns*, non-significant.

Supplementary Tables

Supplementary Table 1. List of plasmids used in this study

CDS	Vector	CDS	Vector
3×Flag-PTEN	pQCXIN	3×Flag-FBXO36	pBabe
3×Flag-PTEN^{NES}	pQCXIN	3×Flag-FBXO44	pBabe
3×Flag-PTEN^{K221R}	pQCXIN	PTEN	pEGFP-N1
3×Flag-PTEN^{K223R}	pQCXIN	PTEN^{K221R}	pEGFP-N1
3×Flag-PTEN^{K237R}	pQCXIN	PTEN^{K13,289E}	pEGFP-N1
3×Flag-PTEN$^{Mut\ A}$	pQCXIN	PTEN$^{1\text{-}351}$	pEGFP-N1
3×Flag-PTEN$^{Mut\ B}$	pQCXIN	PTEN$^{1\text{-}365}$	pEGFP-N1
3×Flag-PTEN$^{Mut\ C}$	pQCXIN	PTEN$^{1\text{-}375}$	pEGFP-N1
3×Flag-PTEN$^{Mut\ D}$	pQCXIN	PTEN$^{\Delta 366\text{-}375}$	pEGFP-N1
3×Flag-PTEN$^{Mut\ B1}$	pQCXIN	PTEN^{4A}	pEGFP-N1
3×Flag-PTEN$^{Mut\ B2}$	pQCXIN	PTEN$^{4A\Delta 366\text{-}375}$	pEGFP-N1
3×Flag-PTEN$^{Mut\ B3}$	pQCXIN	PTEN	pEF-HA
3×Flag-PTEN$^{1\text{-}351}$	pQCXIN	FBXO22^{mNLS}	pEGFP-C1
3×Flag-PTEN$^{1\text{-}365}$	pQCXIN	WWP1	pEGFP-C1
3×Flag-PTEN$^{1\text{-}375}$	pQCXIN	WWP2	pEGFP-C1
3×Flag-PTEN^{4A}	pQCXIN	NEDD4-1	pEGFP-C1
3×Flag-PTEN$^{\Delta 366\text{-}375}$	pQCXIN	FBXO22	pEGFP-C1
3×Flag-PTEN$^{4A\Delta 366\text{-}375}$	pQCXIN	FBXO22ΔF	PLVX-IRES-ZsGREEN1
HA-ubiquitin	pQCXIN	FBXO22	PLVX-IRES-ZsGREEN1
HA-ubiquitin	pCDNA3.1	FBXO22-m5$	PLVX-IRES-ZsGREEN1
3×Flag-CUL1	pFlag-CMV4	CDH1	PLVX-IRES-Puro
PTEN	pBabe	FBXO22^{mNLS} #	pINUCER21
PTEN^{K221R}	pBabe	FBXO22	pINUCER21
3×Flag-FBXO22	pBabe	pTWT*	
3×Flag-FBXO22ΔF	pBabe	pTN24&	
3×Flag-FBXO28	pBabe		

$ A shRNA-resistant version of FBXO22
# The NLS of FBXO22 (K^{121}RARKR^{126}) was mutated to mNLS (A^{121}AARAA^{126})
* pTWT was a gift from Christopher W. J. Smith (University of Cambridge, Cambridge, UK) [1]
& pTN24 was a gift from Ian C. Eperon (University of Leicester, Leicester, UK) [2]

Supplementary Table 2. Target sequences of shRNAs and gRNAs used in this study

shRNA	Vector	Target sequence
shFBXO22	pSIREN	GTGTGGTCCTTGTCTTTGGTT
shNEDD4-1	pSIREN	CCGGAGAATTATGGGTGTCAA
shWWP2	pSIREN	CCCAAGGTGCATAATCGTCAA
CRISPR-CAS9		
gRNA	Vector	Target sequence
gFBXO22	pLenti-U6-gRNA-mCMV-SaCa	GACCCGCGGAGCACCTTCGTG

gPTEN	s9-P-2A-sfGFP pLenti-U6-spgRNA v2.0-CMV-Puro-P2A-3Flag-sp Cas9	ACCGGAAATCCCATAGCAATAATGT

Supplementary Table 3. List of antibodies and reagents used in this study

**Western Blot**				
Antibody	Company name	Catalogue number	Dilution	Clone number
SKP1	Cell Signaling Technology	Cat# 12248	1:1000	D3J4N
PLK1	Cell Signaling Technology	Cat# 4513	1:1000	208G4
Akt (pan)	Cell Signaling Technology	Cat# 4691	1:1000	C67E7
Phospho-Akt (Ser 473)	Cell Signaling Technology	Cat# 4060	1:1000	D9E
Phospho-Akt (Thr 308)	Cell Signaling Technology	Cat# 4056	1:1000	244F9
PTEN	Cell Signaling Technology	Cat# 9559	1:1000	138G6
Phospho-PTEN (Ser 308/Thr 382/Thr 383)	Cell Signaling Technology	Cat# 9554S	1:1000	
Aurora A	Cell Signaling Technology	Cat# 12100	1:1000	1F8
PARP	Cell Signaling Technology	Cat# 9532	1:1000	46D11
Ubiquitin	Cell Signaling Technology	Cat# 3933	1:1000	D9D5
K48-linkage Specific Polyubiquitin	Cell Signaling Technology	Cat# 8081S	1:1000	
K63-linkage Specific Polyubiquitin	Millipore	Cat# 05-1308	1:1000	
Cyclin A2	Proteintech	Cat# 18202-1-AP	1:1000	
FBXO22	Proteintech	Cat# 13606-1-AP	1:1000	
CUL1	Proteintech	Cat# 12895-1-AP	1:1000	
BACH1	Proteintech	Cat# 14018-1-AP	1:1000	
NEDD4-1	Santa Cruz	Cat# sc25508	1:1000	
WWP2	Santa Cruz	Cat# sc30052	1:1000	
GFP	abcam	Cat# ab183734	1:1000	EPR14104
Lamin-B1	abcam	Cat# ab133741	1:1000	EPR8985B
HA	Sigma-Aldrich	Cat# H6908	1:1000	
FLAG-HRP	Sigma-Aldrich	Cat# A8592	1:1000	
β-tubulin	Sigma-Aldrich	Cat# T4026	1:2000	
CDH1	Life Technologies	Cat# 34-2000	1:500	
β-actin	MBL	Cat# PM053-7	1:5000	
Anti-mouse IgG, HRP-linked Antibody	Cell Signaling Technology	Cat# 7076	1:2000	
Anti-rabbit IgG, HRP-linked Antibody	Cell Signaling Technology	Cat# 7074	1:2000	
**Immunofluorescence**				
Antibody	Company name	Catalogue number	Dilution	

NEDD4-1	Santa Cruz	Cat# sc25508	1:100	
WWP2	Santa Cruz	Cat# sc30052	1:100	
Flag-tag	Sigma-Aldrich	Cat# F3040	1:200	
p230	BD Transduction Laboratories	Cat# 611280	1:100	
**Immunohistochemistry**				
Antibody	Company name	Catalogue number	Dilution	
PTEN	Millipore	Cat# 04-035	1:100	26H9
FBXO22	Proteintech	Cat# 13606-1-AP	1:200	
**Immunoprecipitation**				
Antibody	Company name	Catalogue number		
anti-Flag M2 beads	Sigma	Cat# A2220		
anti-GFP Agarose	MBL	Cat# 153-8		
PTEN	Cell Signaling Technology	Cat# 9556		
**Reagent**	Company name	Catalogue number		
Cycloheximide (CHX)	Biovision	Cat# 1041		
MG132	Calbiochem	Cat# 133407-82-6		
Bortezomib (PS-341)	Selleck	Cat# S1013		
Brefeldin A (BFA)	Selleck	Cat# S7046		
NH_4Cl	Sigma-Aldrich	Cat# 254134		

Supplementary Table 4. List of primers used in quantitative real-time PCR

qRT-PCR		
Gene Name	Forward (5' to 3')	Reverse (5' to 3')
FBXO22	GTGTTGAGTAACCTGGCGG	TGCGAACATTCTCAAGCTCCT
$pTWT^{1\text{-}3\text{-}4}$	ACTGGAGCTGGCGGAGAAAAA	ACTCACTGCGTTCCAGGCAATGCT
$pTWT^{1\text{-}4}$	CGGAGCAAGCAGGCTGAAGCT	ACTCACTGCGTTCCAGGCAATGCT
$GOLGA2^{L}$	CAGCAGAGGAATAGCCCTGG	AACGGTTAAGGTCGGACACC
$GOLGA2^{S}$	TGTCGGAAGAAACCCGACAG	TCCTTGGGTGTATCCTCAGGT
18S RNA	GAGAGTGAGCGGCAGAGC	GCTCCCAAGATCCAACTACGAG

Supplementary References

1. Gromak, N. & Smith, C.W. A splicing silencer that regulates smooth muscle specific alternative splicing is active in multiple cell types. *Nucleic Acids Res* **30**, 3548-3557 (2002).
2. Nasim, M.T. & Eperon, I.C. A double-reporter splicing assay for determining splicing efficiency in mammalian cells. *Nat Protoc* **1**, 1022-1028 (2006).
