# Supplementary material for: FBXO22 degrades nuclear PTEN to promote tumorigenesis
Source: Nat Commun. 2020 Apr 6;11:1720. doi: 10.1038/s41467-020-15578-1 (PMC7136256; doi:10.1038/s41467-020-15578-1)
Supplement: Supplementary file 3 — Description of Additional Supplementary Files [file 41467_2020_15578_MOESM3_ESM.pdf]

Description of Additional Supplementary Files

File Name: Supplementary Data 1
Description: List of PTEN interacting proteins

File Name: Supplementary Data 2
Description: Information on samples of colorectal cancer patients

File Name: Supplementary Data 3
Description: Information on tissue microarray of colon cancer patients
