# Supplementary material for: FBXO22 degrades nuclear PTEN to promote tumorigenesis
Source: Nat Commun. 2020 Apr 6;11:1720. doi: 10.1038/s41467-020-15578-1 (PMC7136256; doi:10.1038/s41467-020-15578-1)
Supplement: Supplementary file 7 — Reporting Summary [file 41467_2020_15578_MOESM7_ESM.pdf]

Corresponding author(s): Shao-Ming Shen
Last updated by author(s): Feb 27, 2020

Reporting Summary

Nature Research wishes to improve the reproducibility of the work that we publish. This form provides structure for consistency and transparency in reporting. For further information on Nature Research policies, see Authors & Referees and the Editorial Policy Checklist.

Statistics

For all statistical analyses, confirm that the following items are present in the figure legend, table legend, main text, or Methods section.

n/a	Confirmed	
☐	☒	The exact sample size (n) for each experimental group/condition, given as a discrete number and unit of measurement
☐	☒	A statement on whether measurements were taken from distinct samples or whether the same sample was measured repeatedly
☐	☒	The statistical test(s) used AND whether they are one- or two-sided<br>*Only common tests should be described solely by name; describe more complex techniques in the Methods section.*
☒	☐	A description of all covariates tested
☒	☐	A description of any assumptions or corrections, such as tests of normality and adjustment for multiple comparisons
☐	☒	A full description of the statistical parameters including central tendency (e.g. means) or other basic estimates (e.g. regression coefficient) AND variation (e.g. standard deviation) or associated estimates of uncertainty (e.g. confidence intervals)
☐	☒	For null hypothesis testing, the test statistic (e.g. F, t, r) with confidence intervals, effect sizes, degrees of freedom and P value noted<br>*Give P values as exact values whenever suitable.*
☒	☐	For Bayesian analysis, information on the choice of priors and Markov chain Monte Carlo settings
☒	☐	For hierarchical and complex designs, identification of the appropriate level for tests and full reporting of outcomes
☒	☐	Estimates of effect sizes (e.g. Cohen's d, Pearson's r), indicating how they were calculated

Our web collection on statistics for biologists contains articles on many of the points above.

Software and code

Policy information about availability of computer code

Data collection	Software Image J was used for quantification of immunoblot intensity. Flow cytometric data was analyzed by FlowJo Version 10. Microsoft Excel 2016, GraphPad Prism 7 (GraphPad Software) and IBM SPSS Statistics 20 were used to analyze statistical data, GraphPad Prism7 was used to draw graphs in the study, Microsoft PowerPoint 2016 and Photoshop CS6 was used to crop images from unprocessed images.
Data analysis	Mass Spectrometry: ProteoWizard msConvert, Mascot (Matrix Science, London, UK; version 2.4.1), Scaffold (version Scaffold_4.2.1, Proteome Software Inc., Portland, OR); Gene set scores were correlated with patient survival data using the coxph function in the Survival R package; The RNA expression data were retrieved from public datasets: TCGA cohorts.

For manuscripts utilizing custom algorithms or software that are central to the research but not yet described in published literature, software must be made available to editors/reviewers. We strongly encourage code deposition in a community repository (e.g. GitHub). See the Nature Research guidelines for submitting code & software for further information.

Data

Policy information about availability of data

All manuscripts must include a data availability statement. This statement should provide the following information, where applicable:
- Accession codes, unique identifiers, or web links for publicly available datasets
- A list of figures that have associated raw data
- A description of any restrictions on data availability

The data supporting the findings of this manuscript are available from the corresponding author upon reasonable request. Previously published RNA-Seq data that were reanalyzed here are available under accession code GSE17536. The source data underlying Figs. 1b-d, 2a-i, 3a-c, 3e-g, 4a-e, 4g-j, 5a-e, 6a-f, 7c, 7f and supplementary Figs. 1a, 1d, 2a, 2b, 2d, 2f, 2h, 3a-d, 4a-g, 5b-d are provided as a Source Data file.

Field-specific reporting

Please select the one below that is the best fit for your research. If you are not sure, read the appropriate sections before making your selection.

☒ Life sciences ☐ Behavioural & social sciences ☐ Ecological, evolutionary & environmental sciences

For a reference copy of the document with all sections, see nature.com/documents/nr-reporting-summary-flat.pdf

Life sciences study design

All studies must disclose on these points even when the disclosure is negative.

Sample size	No statistical methods were used to predetermine the sample size. Required sample sizes were estimated based on our experience performing similar experiments in previous publications. Sample sizes are stated in figure legends.
Data exclusions	No data were excluded from the analyses.
Replication	Experiments were performed in at least two independent biological replicates unless indicated specifically. For most experiments, mean and standard deviation or the individual results of all replicates are shown. For western blots, immunofluorescence and immunohistochemistry, representative results were shown. Replication was successful in all attempts.
Randomization	The clones used for individual experiment were picked randomly from a cultural streak for single colony.<br>Human colorectal cancer samples were randomly selected from human tissue bank of Ruijin Hospital of Shanghai Jiao Tong University School of Medicine (SJTU-SM).<br>In other experiments, cells, samples and mice were also randomly allocated into experimental groups.
Blinding	Investigators were blinded during data collection and analysis.

Reporting for specific materials, systems and methods

We require information from authors about some types of materials, experimental systems and methods used in many studies. Here, indicate whether each material, system or method listed is relevant to your study. If you are not sure if a list item applies to your research, read the appropriate section before selecting a response.

Materials & experimental systems

n/a	Involved in the study	
☐	☒	Antibodies
☐	☒	Eukaryotic cell lines
☒	☐	Palaeontology
☐	☒	Animals and other organisms
☐	☒	Human research participants
☒	☐	Clinical data

Methods

n/a	Involved in the study	
☒	☐	ChIP-seq
☐	☒	Flow cytometry
☒	☐	MRI-based neuroimaging

Antibodies

Antibodies used

Antibodies used for western blot (supplier name, catalog number, dilution, clone name):
SKP1 Cell Signaling Technology Cat# 12248 1:1000 D3J4N;
PLK1 Cell Signaling Technology Cat# 4513 1:1000 208G4;
Akt (pan) Cell Signaling Technology Cat# 4691 1:1000 C67E7;
Phospho-Akt (Ser 473) Cell Signaling Technology Cat# 4060 1:1000 D9E;
Phospho-Akt (Thr 308) Cell Signaling Technology Cat# 4056 1:1000 244F9;
PTEN Cell Signaling Technology Cat# 9559 1:1000 138G6;
Phospho-PTEN (Ser 308/Thr 382/Thr 383) Cell Signaling Technology Cat# 9554S 1:1000;
Aurora A Cell Signaling Technology Cat# 12100 1:1000 1F8;
PARP Cell Signaling Technology Cat# 9532 1:1000 46D11;
Ubiquitin Cell Signaling Technology Cat# 3933 1:1000;
K48-linkage Specific Polyubiquitin Cell Signaling Technology Cat# 8081S 1:1000 D9D5;
K63-linkage Specific Polyubiquitin Millipore Cat# 05-1308 1:1000;
Cyclin A2 Proteintech Cat# 18202-1-AP 1:1000;
FBXO22 Proteintech Cat# 13606-1-AP 1:1000;
CUL1 Proteintech Cat# 12895-1-AP 1:1000;
BACH1 Proteintech Cat# 14018-1-AP 1:1000;
NEDD4-1 Santa Cruz Cat# sc25508 1:1000;
WWP2 Santa Cruz Cat# sc30052 1:1000;
GFP abcam Cat# ab183734 1:1000 EPR14104;

Lamin-B1 abcam Cat# ab133741 1:1000 EPR8985B;
HA Sigma-Aldrich Cat# H6908 1:1000;
FLAG-HRP Sigma-Aldrich Cat# A8592 1:1000 M2;
b-tubulin Sigma-Aldrich Cat# T4026 1:2000;
CDH1 Life Technologies Cat# 34-2000 1:500
b-actin MBL Cat# PM053-7 1:5000;
Anti-mouse IgG, HRP-linked Antibody Cell Signaling Technology Cat# 7076 1:2000
Anti-rabbit IgG, HRP-linked Antibody Cell Signaling Technology Cat# 7074 1:2000

Antibodies used for Immunofluorescence:
NEDD4-1 Santa Cruz Cat# sc25508 1:100;
WWP2 Santa Cruz Cat# sc30052 1:100;
Flag-tag Sigma-Aldrich Cat# F3040 1:200
p230 BD Transduction Laboratories Cat# 611280 1:100

Antibodies used for Immunohistochemistry:
PTEN Millipore Cat# 04-035 1:100 6H2.1;
FBXO22 Proteintech Cat# 13606-1-AP 1:200

Antibodies used for Immunoprecipitation:
anti-Flag M2 beads Sigma Cat# A2220;
anti-GFP Agarose MBL Cat# 153-8;
PTEN Cell Signaling Technology Cat# 9556 26H9;

Validation

SKP1 https://www.cellsignal.com/products/primary-antibodies/skp1-d3j4n-rabbit-mab/12248
PLK1 https://www.cellsignal.com/products/primary-antibodies/plk1-208g4-rabbit-mab/4513
Akt (pan) https://www.cellsignal.com/products/primary-antibodies/akt-pan-c67e7-rabbit-mab/4691
Phospho-Akt (Ser 473) https://www.cellsignal.com/products/primary-antibodies/phospho-akt-ser473-d9e-xp-rabbit-mab/4060
Phospho-Akt (Thr 308) https://www.cellsignal.com/products/primary-antibodies/phospho-akt-thr308-244f9-rabbit-mab/4056
PTEN https://www.cellsignal.com/products/primary-antibodies/pten-138g6-rabbit-mab/9559
Phospho-PTEN (Ser 308/Thr 382/Thr 383) https://www.cellsignal.com/products/primary-antibodies/phospho-pten-ser380-thr382-383-antibody/9554
Aurora A https://www.cellsignal.com/products/primary-antibodies/aurora-a-1f8-mouse-mab/12100
PARP https://www.cellsignal.com/products/primary-antibodies/parp-46d11-rabbit-mab/9532
Ubiquitin https://www.cellsignal.com/products/primary-antibodies/ubiquitin-antibody/3933
K48-linkage Specific Polyubiquitin https://www.cellsignal.com/products/primary-antibodies/k48-linkage-specific-polyubiquitin-d9d5-rabbit-mab/8081
K63-linkage Specific Polyubiquitin Millipore https://www.merckmillipore.com/CN/zh/product/Anti-Ubiquitin-Antibody-Lys63-Specific-clone-Apu3-rabbit-monoclonal,MM_NF-05-1308?ReferrerURL=https%3A%2F%2Fcn.bing.com%2F&bd=1
Cyclin A2 https://www.ptglab.com/Products/CCNA2-Antibody-18202-1-AP.htm
FBXO22 https://www.ptglab.com/Products/FBXO22-Antibody-13606-1-AP.htm
CUL1 https://www.ptglab.com/products/CUL1-Antibody-12895-1-AP.htm
BACH1 https://www.ptglab.com/products/BACH1-Antibody-14018-1-AP.htm
GFP https://www.abcam.com/gfp-antibody-epr14104-ab183734.html
Lamin-B1 https://www.abcam.com/Lamin-B1-antibody-EPR8985B-ab133741.html
HA https://www.sigmaaldrich.com/catalog/product/sigma/h6908?lang=en&region=US
FLAG- https://www.sigmaaldrich.com/catalog/product/sigma/a8592
b-tubulin https://www.sigmaaldrich.com/catalog/product/sigma/t4026
CDH1 https://www.thermofisher.com/cn/en/antibody/product/FZR1-Antibody-Polyclonal/34-2000
b-actin http://ruo.mbl.co.jp/bio/dtl/A/?pcd=PM053
Anti-mouse IgG, HRP-linked Antibody https://www.cellsignal.com/products/secondary-antibodies/anti-mouse-igg-hrp-linked-antibody/7076
Anti-rabbit IgG, HRP-linked Antibody https://www.cellsignal.com/products/secondary-antibodies/anti-rabbit-igg-hrp-linked-antibody/7074
Flag-tag https://www.sigmaaldrich.com/catalog/product/sigma/f3040
p230 https://www.bdbiosciences.com/us/reagents/research/antibodies-buffers/cell-biology-reagents/cell-biology-antibodies/purified-mouse-anti-human-p230-trans-golgi-15p230-trans-golgi/p/611280
PTEN http://www.merckmillipore.com/CN/zh/product/Anti-PTEN-Antibody-clone-6H2.1,MM_NF-04-035
PTEN https://www.cellsignal.com/products/primary-antibodies/pten-26h9-mouse-mab/9556

Eukaryotic cell lines

Policy information about cell lines

Cell line source(s)

Mouse embryonic fibroblasts (MEF) were isolated from mouse embryo.

Other cell lines were purchased from Cell Bank of Chinese Academy of Sciences, Shanghai.
Human embryonic kidney HEK293T (293T);
human cervical cancer Hela;
human colorectal cancer SW620;
human colorectal cancer SW480;
human colorectal cancer LS174T;

Authentication	Cell line authentication was performed via short tandem repeat profiling.
Mycoplasma contamination	There were no signs of mycoplasma contamination for all cell lines.
Commonly misidentified lines (See ICLAC register)	No ICLAC samples were used.

Animals and other organisms

Policy information about studies involving animals; ARRIVE guidelines recommended for reporting animal research

Laboratory animals	4-6 weeks female nude mice and NOD-SCID mice were used to inoculate subcutaneously. Mice were housed in groups (3-5 per cage) at 22-24 °C with a 12 h light-dark cycle and ad libitum access to regular chow diet and water.
Wild animals	No wild animals were used.
Field-collected samples	No field-collected samples were used.
Ethics oversight	Animal care and experiments were compliant with all of the relevant ethical regulations regarding animal research and were approved by the committee for humane treatment of animals at Shanghai Jiao Tong University School of Medicine.

Note that full information on the approval of the study protocol must also be provided in the manuscript.

Human research participants

Policy information about studies involving human research participants

Population characteristics	Clinical human colorectal cancer used in qRT-PCR analysis were histopathologically and clinically diagnosed. Among the cohort of 37 patients, there are 27 male and 10 female patients, including 14 colon cancer patients, 21 rectum cancer patients and 2 multiple tumor patients. The details of colorectal cancer patients were provided in Supplementary Table 2. Clinical human colon cancer used in microarray analysis were histopathologically and clinically diagnosed. Among the cohort of 72 colon colon cancer patients, there are 41 male and 31 female patients, 5 in stage I, 41 in stage II, 22 in stage III, 3 in stage IV and one case had unavailable information for pathological stage. The details of patients were provided in Supplementary Table 3.
Recruitment	Human colorectal cancer samples for qRT-PCR analysis were randomly selected with informed consent from human tissue bank of Ruijin Hospital of Shanghai Jiao Tong University School of Medicine (SJTU-SM). Tissue microarray of human colorectal tumors and paired adjacent normal tissues were purchased from Shanghai Outdo Biotech Co (Shanghai, China). The selection of patients was random and we believe that there was no bias in data selection.
Ethics oversight	All experiments from human samples were performed with the approval of the Medical Ethic Committee of Shanghai JiaoTong University School of Medicine.

Note that full information on the approval of the study protocol must also be provided in the manuscript.

Flow Cytometry

Plots

Confirm that:

☐ The axis labels state the marker and fluorochrome used (e.g. CD4-FITC).

☐ The axis scales are clearly visible. Include numbers along axes only for bottom left plot of group (a 'group' is an analysis of identical markers).

☐ All plots are contour plots with outliers or pseudocolor plots.

☒ A numerical value for number of cells or percentage (with statistics) is provided.

Methodology

Sample preparation	SW620 and SW480 cells were collected, rinsed and fixed overnight with 75% cold ethanol at -20°C. Cells were then treated with 100 µg/ml RNase A in PBS and stained with 25 µg/ml propidium iodide. Samples were analyzed by flow cytometry for the DNA content.
Instrument	FACSCalibur fow cytometry (BD Biosciences)
Software	Flow cytometry data were recorded by BD CellQuest program and analyzed using the FlowJo version 10 software.
Cell population abundance	No sorting was conducted.

Gating strategy

Main population of single cells were gated in FSC-A/SSC-A. Two major peaks were observed by PI fluorescence, one peak was labelled as G1, another peak was labelled as G2/M and intermediate region was labelled as S phase. Gating parameters were set using negative control samples.

☐ Tick this box to confirm that a figure exemplifying the gating strategy is provided in the Supplementary Information.
